# Correlations between the Signal Complexity of Cerebral and Cardiac Electrical Activity: A Multiscale Entropy Analysis

**DOI:** 10.1371/journal.pone.0087798

**Published:** 2014-02-03

**Authors:** Pei-Feng Lin, Men-Tzung Lo, Jenho Tsao, Yi-Chung Chang, Chen Lin, Yi-Lwun Ho

**Affiliations:** 1 Department of Geriatrics, Tainan Hospital, Ministry of Health and Welfare, Tainan, Taiwan; 2 Graduate Institute of Biomedical Electronics and Bioinformatics, National Taiwan University, Taipei, Taiwan; 3 Research Center for Adaptive Data Analysis, National Central University, Jongli, Taiwan; 4 Graduate Institute of Communication Engineering, National Taiwan University, Taipei, Taiwan; 5 Institute of Systems Biology and Bioinformatics, National Central University, Jongli, Taiwan; 6 Graduate Institute of Clinical Medicine, College of Medicine, National Taiwan University, Taipei, Taiwan; 7 Cardiology, Department of Internal Medicine, National Taiwan University Hospital, Taipei, Taiwan; Universidad Veracruzana, Mexico

## Abstract

The heart begins to beat before the brain is formed. Whether conventional hierarchical central commands sent by the brain to the heart alone explain all the interplay between these two organs should be reconsidered. Here, we demonstrate correlations between the signal complexity of brain and cardiac activity. Eighty-seven geriatric outpatients with healthy hearts and varied cognitive abilities each provided a 24-hour electrocardiography (ECG) and a 19-channel eye-closed routine electroencephalography (EEG). Multiscale entropy (MSE) analysis was applied to three epochs (resting-awake state, photic stimulation of fast frequencies (fast-PS), and photic stimulation of slow frequencies (slow-PS)) of EEG in the 1–58 Hz frequency range, and three RR interval (RRI) time series (awake-state, sleep and that concomitant with the EEG) for each subject. The low-to-high frequency power (LF/HF) ratio of RRI was calculated to represent sympatho-vagal balance. With statistics after Bonferroni corrections, we found that: (a) the summed MSE value on coarse scales of the awake RRI (scales 11–20, RRI-MSE-coarse) were inversely correlated with the summed MSE value on coarse scales of the resting-awake EEG (scales 6–20, EEG-MSE-coarse) at Fp2, C4, T6 and T4; (b) the awake RRI-MSE-coarse was inversely correlated with the fast-PS EEG-MSE-coarse at O1, O2 and C4; (c) the sleep RRI-MSE-coarse was inversely correlated with the slow-PS EEG-MSE-coarse at Fp2; (d) the RRI-MSE-coarse and LF/HF ratio of the awake RRI were correlated positively to each other; (e) the EEG-MSE-coarse at F8 was proportional to the cognitive test score; (f) the results conform to the cholinergic hypothesis which states that cognitive impairment causes reduction in vagal cardiac modulation; (g) fast-PS significantly lowered the EEG-MSE-coarse globally. Whether these heart-brain correlations could be fully explained by the central autonomic network is unknown and needs further exploration.

## Introduction

The brain-heart connection remains not fully understood. Conventionally, the heart and brain are believed to be connected in a hierarchical way that the heart receives the brain’s commands through the central autonomic network [1], where the prefrontal cortex (mainly the right side) plays the leading role. Thayer et al. proposed a neurovisceral integration model to account for the linkage between the cognitive-affective processing system and the autonomic nervous system [2]. A relationship was found between vagal tone and event-related potentials [3], [4]. However, the heart begins to beat before the brain is formed. A transplanted heart can immediately satisfy the physiological demands of its new host without connection to the host’s nervous system. Recent evidence also showed that the intrinsic cardiac ganglia and intrathoracic extracardiac ganglia can process information independently of the brain [5]. We hypothesized that the heart-brain connection conveys more information than just heart rate alone.

Biological systems are complex at multiple levels of temporal and spatial scales and consist of interconnected feedback loops. The Fourier-based spectral analysis averages the signals, so it can not sufficiently display the nonlinear and non-stationary properties of complex biological systems. The science of complex systems is closely related to variability analysis which detects and characterizes nonlinear dynamics [6]. Heart rate variability (HRV) and signal variability of resting-state brain activity convey important information about network dynamics [7]. We found the entropy measurement techniques, which compute the regularity patterns of a time series, best suit our data and the entropy values can provide quantitative connotations to facilitate comparisons and correlations between two systems and between individual subjects.

**Figure 1:**
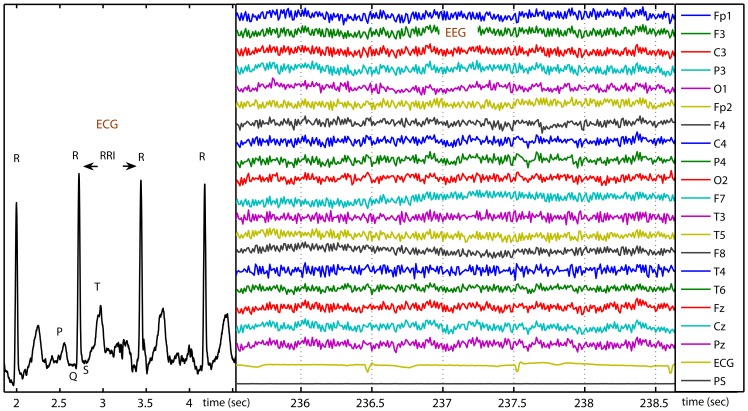
Electrocardiography (ECG) and electroencephalography (EEG). One cycle of ECG includes various deflections, P, Q, R, S (QRS complex) and T. All R peaks of ECG recordings were detected to obtain the RR interval (RRI) time series. Each EEG recording includes brain waves from 19 electrode sites, one ECG recording and one trace of photic stimulation (PS).

The entropy methods include the evaluation of either entropy (Shannon entropy) or entropy rate (approximate entropy (ApEn) [8], sample entropy (SampEn) [9] and multiscale entropy (MSE) [10]). SampEn, without counting self-matches, is less dependent on the signal length and shows more consistency on a broader range of parameters than ApEn [9]. MSE, based on SampEn, takes into account the correlations inherent in biological signals at multiple time scales. Multifractality is present in HRV [11], [12], blood pressure dynamics [13] and electroencephalography (EEG) [14], [15]. Although the MSE analysis was derived from stationary processes, only those non-stationarities on scales much larger than those considered for the MSE analysis may affect the consistency of the results in practice [16]. Successful applications of MSE were seen in studies of HRV [17], [18], human neuronal spiking patterns [19], postural sway patterns [16], and in EEGs of brain maturation [20]–[22], epilepsy [23], aging [24], dementia [25], [26] and schizophrenia [27]. Here, we examined the MSE results of both EEG and RR interval (RRI, R-to-R peak interval of ECG, please see Figure 1) time series together and sought to establish what, if any, relationships exist between the dynamics of cardiac and cerebral electrical activity.

Photic stimulation (PS) is a procedure meant to elicit or accentuate epileptiform discharges during a routine EEG. Both cardiac and neuron cells are spontaneous oscillators. Phase-locked dynamics have been observed in cardiac cells [28] and neurons [29] when they are stimulated by periodic electrical impulses. Mechanical stimulation through ventilator can also produce such phenomenon to neural cells in the respiratory center [30] and sympathetic neurons [31]. This phase-locking phenomenon may not be rigidly fixed as the coupling ratio [32] and phase [31] could be various. The brain is stimulated by periodic lighting impulses during the PS procedure. Despite the widespread utilization, the complete understanding of the brain response to PS is still an open problem. We also checked the signal complexity in the EEGs under repetitive PS.

## Materials and Methods

### Subjects

The final study population included 87 geriatric outpatients, who were free of previously diagnosed cardiovascular (except mild hypertension) and neurologic diseases, and found to have varied cognitive abilities (female  =  42; age  =  79.1±6.4 years, mean ± standard deviation (SD), range 65.3 – 93.7 years). Fifty-eight (female  =  29; age  =  81.0±5.7 years) newly diagnosed cases of dementia presented on the first visit with a chief complaint of memory or cognitive decline, corroborated by informants, and had a Chinese version of the mini-mental state examination, the mini-mental state examination of Taiwan, version 1 (MMSE-T1) score with illiteracy adjustment less than or equal to 26. After laboratory tests and brain-imaging referrals, the recruited demented patients included only two types: probable Alzheimer’s disease (AD) (n = 22; female  =  7, age  =  81.9±6.6 years; MMSE-T1  =  22.2±5.8) according to NINCDS-ADRDA [33] and vascular dementia (VD) (n = 36; female  =  22, age  =  80.4±5.2 years; MMSE-T1  =  18.4±7.2) of *s*ubcortical arteriosclerotic encephalopathy according to NINDS-AIREN [34]. The control group consisted of Twenty-nine (female  =  13; 75.5±6.2 years; MMSE-T1  =  28.4±0.9) ambulatory geriatric patients with only mild hypertension and/or mild diabetes. The original MMSE-T1 scores were adjusted for illiteracy by multiplying 30/27 (3 points for reading or writing Chinese characters). Exclusion criteria included mixed dementia, heart failure, atrial fibrillation, frequent atrial premature complex or ventricular premature complex, major systemic diseases, infection, hypothyroidism, vitamin B12 or folic acid deficiency, psychosis, previous stroke, major head injury, epilepsy, normal pressure hydrocephalus, subdural effusion or hemorrhage, and exposure to sympatholytic agents (including beta blockers), acetyl cholinesterase inhibitors, tranquilizers or antidepressants.

### Ethics statement

The ethics committee on human research of Tainan hospital approved the study (IRB-2008004). All participants or their surrogates gave written informed consent. The investigation conformed to the principles of the Declaration of Helsinki.

### Data collection

All subjects underwent electrocardiography (ECG) monitoring for 24 hours by a standard ambulatory ECG recorder (MyECG E3-80 Portable Recorder, Microstar, Taiwan) at 250 Hz. Two epochs of 2-hour ECG recorded during 9–11am (awake) and 1–3 am (sleep) were obtained from each subject. Another 7-minute ECG recording was extracted from the resting-awake EEG for each subject (Figure 1). The R-peak detection was performed by an automated arrhythmia detection algorithm and corrected by visual inspection. Occasional ectopic beats were identified and replaced with linearly interpolated RRI data. Those people with a rate of ectopic beats higher than 1 % were excluded from the final analysis. Four people having too many ectopic beats only during sleep were included in the final analysis without the sleep RRI data. The three RRI time series were linearly resampled at 2 Hz. Because of insufficient data points for the 7-minute RRI, only the two 2-hour RRI time series proceeded to the MSE analysis.

All subjects underwent routine EEG recordings with references at ear electrodes within 3 days after the ECG procedure. The routine EEG includes two parts: the 30-minute resting-awake recording and the 2.5-minute recording under intermittent photic stimulation. The surface EEG was collected by a digital EEG recorder (Harmonie version 3.1 digital EEG Stellate Systems, Canada) at 200 Hz from the 19 electrodes of the international standard 10/20 system (Figure 1). The raw data, contaminated with artifacts such as eye movements, blinks, muscle activities and others, were saved in text files for off-line analysis on a personal computer. We chose three 80-second segments from each file: one visually-censored (by an experienced neurologist) artifact-free eye-closed resting-awake recording, one photic-simulated recording at frequency 1, 3, 6 and 9 Hz (slow-PS, duration 10 seconds and interval 10 seconds) and one photic-simulated recording at frequency 12, 15, 18 and 24 Hz (fast-PS, duration 10 seconds and interval 10 seconds). The segments were firstly processed by a notch filter at the frequency of current 60 Hz before further processing.

### Multiscale entropy (MSE)

The method of MSE analysis [10], [18] inspects signals at different time scales by performing the coarse-graining procedure. The process of coarse-graining in MSE is: given a one-dimensional discrete time series, {*X*_1_,…, *X_i_*,…, *X_N_*}, construct consecutive coarse-grained time series, {*y* ^(^ 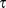 ^)^}, determined by the scale factor, 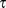, according to the equation: 
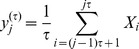
, 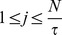. For scale one, the time series {*y* ^(1)^} is simply the original time series. The length of each coarse-grained time series is *N*/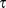. The sample entropy (SampEn) for each coarse grained time series is measured and plotted as a function of the scale factor. To describe SampEn in short, when *m*, *r* and *N*, referred to as pattern length, normalized threshold (normalized by the standard deviation of the original sequence), and signal length respectively, suppose *B_m_* (*r*) is the probability that two sequences will match for *m* points, and *A_m_* (*r*) is the probability that two sequences will match for *m* + 1 points. The match is considered within tolerance and with self-matches excluded. The parameter, SampEn, is estimated by the statistic SampEn (*m*, *r*, *N*)  = – ln [*A_m_* (*r*)/*B_m_* (*r*)]. According to previous studies [10], [35], we calculated SampEn with the parameters *m* = 2, *r* = 0.15 in the range 1 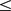 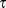 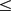 20. Our results were found to be robust against the choices of *m* and *r*. Because the MSE method is sensitive to very low frequency noises, we eliminated the RRI frequency components below 0.00056 Hz and extracted the EEG components in the 1–58 Hz frequency range using the empirical mode decomposition (EMD) technique [36], [37]. Each EEG segment was down-sampled to 100 Hz for the MSE analysis in order to be close in time-scales to the RRI time series.

### Empirical mode decomposition (EMD)

The decomposition is based on the simple assumption that any data consists of a finite number of intrinsic modes of oscillations. For a time series *x* (*t*) with at least 2 extremes, the EMD applies a sifting procedure to extract intrinsic mode functions (IMFs) one by one from a smallest to the largest time scale.
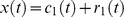


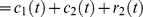


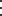





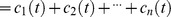



where *c_k_* (*t*) is the *k*th IMF and 
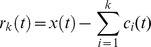
 is the residual after extracting the first *k* IMFs. The steps of sifting process to extract the *k*th IMF [38]:

(1) Initialize *h*_0_ (*t*)  = *h_i_*_−1_ (*t*)  = *r_k_*_−1_ (*t*) (if *k* = 1, *h*_0_ (*t*)  = *x* (*t*)), where *i* = 1;

(2) Extract local minima and maxima of *h_i_*_−1_ (*t*) (if the total number of minima and maxima is less than 2, *c_k_* (*t*)  = *h_i_*_−1_ (*t*) and it’s the end of the whole EMD process);

(3) Obtain upper envelope, *u* (*t*), and lower envelope, *l* (*t*), by the cubic spline interpolation for local minima and maxima of *h_i_*_−1_ (*t*), respectively;

(4) Calculate the *h_i_* (*t*)  = *h_i_*_−1_ (*t*) - mean of (*u* (*t*) + *l* (*t*));

(5) Calculate the standard deviation (SD) of the mean of (*u* (*t*) + *l* (*t*));

(6) To determine a criterion for the sifting process to stop, calculate the limiting size of standard deviation to guarantee that the IMF components retain enough physical sense of both amplitude and frequency modulations.


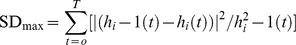
 (typically between 0.2 and 0.3) [37]

(7) When SD < SD_max_, the *k*th IMF is assigned as *c_k_* (*t*)  = *h_i_* (*t*) and *r_k_* (*t*)  = *r_k_*_−1_ (*t*) − *c_k_* (*t*); otherwise repeat steps (2) to (5) for *i* + 1 until SD < SD_max_.

### Statistical analysis

All statistical analyses were performed using R 2.11.0 at a 0.05 alpha level. We used Bonferroni corrections to adjust *p*-values by multiplying the number of the EEG channels (19 channels). Kolmogorov-Smirnov and Levene tests were used to assess the normality of distribution and homoscedasticity, respectively. We used Student’s t-tests to evaluate group differences, and age- and gender-adjusted Pearson’s partial correlation coefficients to evaluate correlations between any two variables. The correlations among the three RRIs or three EEGs were calculated using paired t-tests.

## Results

**Figure 2:**
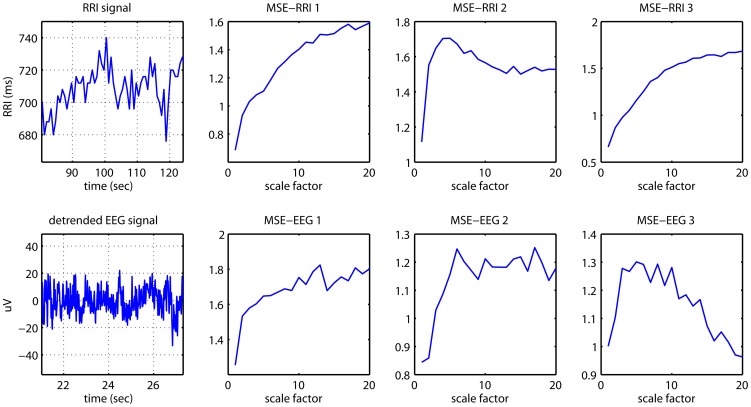
Examples of RR interval (RRI), detrended EEG and the multiscale entropy (MSE) profiles of them. One RRI time series and one detrended single-channel EEG signal are shown on the left-hand side of the figure. MSE-RRI 1-3 are examples of the MSE profiles showing the MSE values of RRI from scale 1 to scale 20, while MSE-EEG 1-3 are examples of the MSE profiles showing the MSE values of EEG from scale 1 to scale 20. All the MSE-profiles show an initial increasing before a plateau or a fall.

We performed a visual inspection of the obtained MSE curves which represent the SampEn values of each coarse-grained sequence versus the scale. Most of the MSE curves had a pattern of an initial increase (from scale 1 to 5 for EEG and from scale 1 to 10 for RRI) before a plateau or a fall. If the SampEn increases initially because of decorrelation before it begins to decrease because of averaging process, the presence of complex long time correlations is expected [38] (Figure 2). We also analyzed regression coefficients for the MSE slopes over 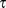 of 1−5, 6−10, 11−15 and 16−20, and found no significant differences between groups. The MSE profiles of either the RRIs or EEGs showed no preference to evolve into a plateau or a fall in either the VD, AD or control subjects. Nevertheless the plateau on the MSE profiles of the EEGs seemed to be higher in the control than in the two demented groups.

**Figure 3:**
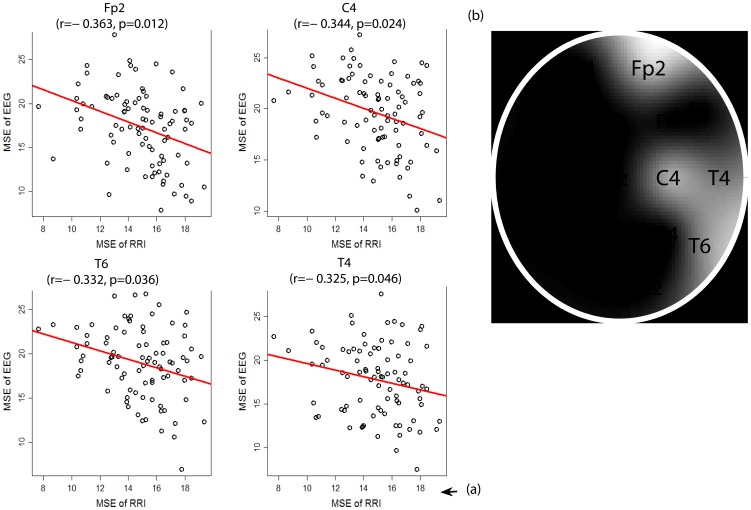
Inverse association between the multiscale entropy (MSE) values of the awake RRI and resting-awake EEG. (a) Regions with significant inverse correlation between the summed MSE values on the scales 11−20 of the awake RRI and the summed MSE values on the scales 6−20 of the resting-awake EEG after Bonferroni corrections (corrected *p*-values  =  original *p*-values
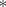
19, alpha  =  0.05). *r* and *p* denote the Pearson’s partial correlation coefficient and corrected significance level, respectively. (b) The brain map illustrates regions with significant association. The relative brightness is according to the sequential p-values from the smallest one (Fp2, C4, T6 and T4).

**Figure 4:**
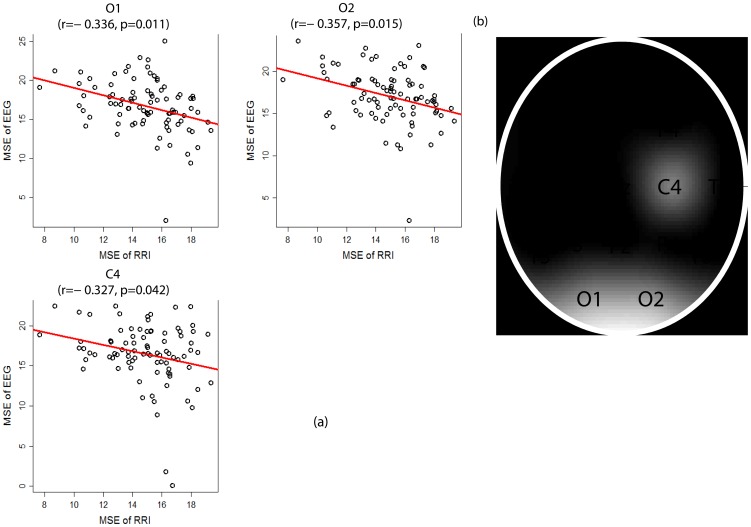
Inverse association between the multiscale entropy (MSE) values of the awake RRI and fast-PS EEG. (a) Regions with significant inverse correlation between the summed MSE values on the scales 11−20 of the awake RRI and the summed MSE values on the scales 6−20 of the photic-simulated EEG at frequency 12, 15, 18 and 24 Hz (fast-PS, duration 10 seconds and interval 10 seconds) after Bonferroni corrections (corrected *p*-values  =  original *p*-values
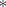
19, alpha  =  0.05). *r* and *p* denote the Pearson’s partial correlation coefficient and corrected significance level, respectively. (b) The brain map illustrates regions with significant association. The relative brightness is according to the sequential p-values from the smallest one (O1, O2 and C4).

In all 87 patients, we found significant and very consistent inverse linear correlations between any of the MSE values of the awake RRIs on the scale from 11 to 20 (after the initial rising) and any of the MSE values of the EEGs in many channels on the scale from 6 to 20 (after the initial rising). Therefore we summed up the MSE values on 10 scales (11−20) for the RRIs and on 15 scales (6−20) for the EEGs to facilitate statistical analyses. Using Pearson’s partial correlation tests with adjustment for age and gender, in all 87 patients, we found significant inverse associations between the summed MSE values on the scales 11−20 of the RRI (RRI-MSE-coarse) during the awake state and the summed MSE values on the scales 6−20 of the EEG (EEG-MSE-coarse) during the resting-awake state after Bonferroni corrections at electrodes Fp2 (*r* = −0.363, *p* = 0.012), C4 (*r* = −0.344, *p* = 0.024), T6 (*r* = −0.332, *p* = 0.036) and T4 (*r* = −0.325, *p* = 0.046) (Figure 3). The inverse associations were present in all three patient groups individually, but failed to reach the alpha level after stringent Bonferroni corrections. The RRI-MSE-coarse of the RRI during sleep was not correlated with the EEG-MSE-coarse of the awake-resting EEG at any channel. The EEG-MSE-coarse of the fast-PS EEG was also inversely correlated to the awake RRI-MSE-coarse after Bonferroni corrections at electrodes O1 (*r* = −0.336, *p* = 0.011), O2 (*r* = −0.357, *p* = 0.015) and C4 (*r* = −0.327, *p* = 0.042) (Figure 4), but not to the sleep RRI-MSE-coarse. In contrast, the EEG-MSE-coarse of the slow-PS EEG was significantly inversely correlated to the sleep RRI-MSE-coarse after Bonferroni corrections at electrode Fp2 (*N* = 83, *r* = −0.332, *p* = 0.049), but not to the awake RRI-MSE-coarse.

In order to examine whether these associations between the complexity of heartbeat and brainwaves come from the autonomic nervous network, we calculated the high frequency power (HF), low frequency power (LF), and ratio of low frequency to high frequency power (LF/HF ratio) for all the three RRI time series. We found that the LF/HF ratio and RRI-MSE-coarse of the awake RRI had a positive age- and gender-adjusted Pearson’s partial correlation coefficient (*r* = 0.307, *p* = 0.004) between each other. Nevertheless, the inverse association between the LF/HF ratio of the awake RRI and the awake-resting EEG-MSE-coarse at any channel was not strong enough to exist after Bonferroni corrections. In contrast, the LF/HF ratio and any of the MSE value on the fine scales (scales 1-3) of the awake RRI were inversely correlated to each other (age- and gender-adjusted Pearson’s partial correlation coefficients *r* = −0.420 to −0.337, *p-*values all < 0.0001). The LF/HF ratio of the sleep RRI was not correlated to the sleep RRI-MSE-coarse or any of the EEG-MSE-coarse. Additionally, we found that both the RRI-MSE-coarse and LF/HF ratio of the awake RRI were negatively correlated to age using gender-adjusted Pearson’s partial correlation tests (*r* = −0.301, *p* = 0.005 and *r* = −0.214, *p* = 0.047, respectively).

Results of Student’s t-tests with Bonferroni corrections revealed that the resting-awake EEG-MSE-coarse at electrode F8 (*p* = 0.036) and the fast-PS EEG-MSE-coarse at electrode Cz (*p* = 0.019) were significantly decreased in the VD group compared to the control group. We also found a significant age- and gender-adjusted Pearson’s partial correlation between the MMSE-T1 score and the resting-awake EEG-MSE-coarse at electrode F8 (*r* = 0.332, *p* = 0.036) after the Bonferroni correction. The resting-awake EEG-MSE-coarse was not correlated to age or gender, whereas the MMSE-T1 score was inversely correlated to age (gender-adjusted Pearson’s partial correlation coefficient *r* = −0.325, *p* = 0.002). The MMSE-T1 scores were significantly lower in the VD than in the AD group using Student’s t-tests (*p* = 0.041). None of the two sets of RRI-MSE-coarse showed group differences among the three patient groups using student’s t-tests after Bonferroni corrections.

The Fourier-based spectra of all three RRI time series were significantly similar to each other in spectral distribution. For the LF, HF and LF/HF ratio between the 2-hour sleep and 2-hour awake RRIs, the *p*-values for Pearson’s correlation coefficients were all below 10^−6^. For the LF and HF between the 7-minute and either of the 2-hour RRIs (awake or sleep), the *p*-values for Pearson’s correlation coefficients were all significantly below 0.001. Of the sleep RRI, the LF and LF/HF ratio (*N* = 83, *p* = 0.003 and 0.019 respectively) were significantly lower in the VD group compared to the control group using Student’s t-tests. In contrast to previous evidence which showed either lower awake LF and LF/HF ratio in AD [39] or no HRV change in AD and VD [40], our patients with VD other than AD had more prominent autonomic cardiac involvement. Finally, the paired-t test also showed that the EEG-MSE-coarse of the fast-PS EEG was much smaller than the EEG-MSE-coarse of either the awake-resting EEG or slow-PS EEG (*p-*values < 0.0001 at all electrode sites).

## Discussion

Our results display inverse correlations between the signal complexity of cardiac and cerebral activities. The central autonomic pathways could not fully explain these correlations. The resting-awake EEG was associated to the awake RRI time series in the right frontopolar, central and temporal area, the fast-PS EEG was also associated to the awake RRI time series in the bilateral occipital and right central area, whereas the slow-PS EEG was associated to the sleep RRI time series in the right frontopolar region. These results may imply a strong correlation between the dynamics of heartbeat and brainwaves; and the correlation could be manipulated by photic stimulation, and affected by the sleep-wake cycle.

A study of EEG under PS found no significant difference between the power spectra of the EEG under PS of frequencies 11 and 20 Hz [41]. We found different signal complexity between the EEGs under different PS frequencies. Compared to the resting-awake EEG, an increase of regularity only occurred with the EEG under PS of frequencies equal and above 12 Hz (fast-PS). The fast-PS procedure made the EEG dynamics much more regular globally and it also shifted the heart-brain associations topographically into the occipital lobes, the visual cortex. The slow-PS procedure, although not causing any obvious change in the signal complexity of EEG, shifted the presence of heart-brain associations from awake-state into sleep. We assume that the stimulation of fast-PS is very strong that highlights the connection between the heart and brain in the visual cortex, whereas the stimulation of slow-PS is weak and only blocks the background activity in the visual cortex just like what happens during sleep, being eye-closed. Sleep is a state of arousable “loss of consciousness” with slowed heartbeats and brainwaves, and the mechanism of sleep remains unknown.

Living organisms are generally believed to behave in a manner of high complexity in order to respond to a broad range of stimuli [42]. With the deterioration of health conditions, the change in dynamic patterns of biological signals is characterized by loss of complexity and development of stereotypy such as Cheyne-Stokes respiration, Parkinsonian gait, cardiac rhythms in heart failure [43] and dementia [44]. Nevertheless, an increase of entropy (ApEn) was noted in the hormone release patterns in Cushing’s disease [45] and acromegaly [46]. This discrepancy may be caused by limitations of the analytic methods or simply imply distinct mechanisms of varied stages or characteristics of the diseases. Vaillancourt and Newell made a point that no one direction fits all results [47]. Any physiological phenomenon plays only one part in the complex networks of a human body. While exploring the dynamics of highly complex physiological signals with a very limited set of signals as state variables, one actually observes a low-dimensional projection of a trajectory embedded in the much higher dimension of state space [18]. Our results, the correlations between the LF/HF ratio and MSE values of the awake RRI being positive on the coarse scales and negative on the fine scales of MSE, advocate the importance of a multiscale approach to biological signals. Riley et al. also revealed that more variability does not mean more randomness, and more controllability does not mean more deterministic characteristics [48]. Therefore the direction of complexity change does not guarantee a better or worse physiological condition. But a consistent inverse correlation most likely indicates a certain physiological connection between the two systems.

Previous evidence showing decreased EEG complexity in dementia only used statistics for group comparison [25], [49], [50], but we found a proportional relationship between the brain signal variability and cognitive test score at electrode F8. Our results correspond with the cholinergic hypothesis which states that cognitive decline (a lower EEG-MSE-coarse) is related to central cholinergic neuronal dysfunction and a consequent decrease in vagal cardiac modulation (a higher LF/HF and a higher RRI-MSE-coarse) [51]. In addition, because of the similarity between all three RRI data, HRV is stable and therefore characteristic of an individual [52]. Finally, conforming to previous evidence, both the MMSE-T1 score and HRV in our study decreased linearly with age.

Although we adopted a stringent statistical criterion by using Bonferroni adjustments to enlarge the *p*-values by 19 times based on the interdependence between the EEGs of 19 electrode sites, we understand that the likelihood of type II error is also increased, so that truly important differences are deemed non-significant [53]. Before Bonferroni corrections, the significant sites showing the heart-brain connection distributed widely over the whole head, whereas after Bonferroni corrections, the heart-brain connection only appeared in the right frontopolar, central and temporal area during the awake state. Whether these correlations between the heart and brain exist globally and favor the right brain, and whether they could be fully explained by the central autonomic pathways are unknown. These correlations seemed to exist in all three aging groups, but whether they exist in younger populations as well is also questionable. According to previous neuroanatomical and pharmacophysiological findings, the prefrontal cortex plays the leading role in the central autonomic network. On the other hand, based on the hypothesis that vagal afferents have diffuse projections into the central nervous system, vagus nerve stimulation can work for refractory epilepsy [54]. The connections between the heart and brain, whether all could be attributed to the autonomic network, are worth further exploration.

There are numerous limitations in this study. A visually clean continuous EEG could only be acquired in a very limited period because of copious artifacts from eye movements, muscles or environments. In this study, we selected visually artifact-free segments from long raw data by an experienced neurologist and excluded the cases who didn’t supply sufficient clean data. The segments were detrended by a deterministic nonlinear method, EMD, based on previous studies [55]. Independent component analysis (ICA), a stochastic approach, can also effectively remove EEG artifacts [56], especially eye-related artifacts [57]. Therefore ICA could have been helpful to treat those excluded cases, of which eye-related artifacts were inevitable. Safieddine et al. compared different methods to remove muscle artifact from EEG data and showed that EMD outperformed ICA for the denoising of data highly contaminated by muscular activity [58]. Finally, the electromagnetic activity of the brain works at an extremely fast speed, and the quasi-stationary epochs of EEG are, in general, short lasting, in the order of tens of seconds [59]. Therefore the simultaneous EEG and ECG data were not long enough for MSE, which warrants long series for better probability estimation.

## Conclusions

The present study demonstrates potential links between the signal complexity of cerebral and cardiac electrical activity for the first time. Life processes demand organ systems to work cooperatively. The source of EEG is still under study ever since the thalamus was emphasized in results of early experiments [60], and so is the origin of heart beat variability. Furthermore, the rapid processing of neural information and the highly efficient changes of cardiac output remain somehow mysterious. A future collection of EEG-ECG pairs from subjects of different age and physical conditions may hopefully provide more information towards a better understanding of the heart-brain connection.

## Supporting Information

Data S1 **This data set includes two 2-hour RRIs of ECG for the VD (case 1-36) and control (case 59-87) groups.** Because the file for the EEG is too big, if someone is interested in obtaining it or the RRIs for the AD group he can contact us via this email address: pflin@hotmail.com.
(RAR)
